# Using genomic epidemiology and geographic activity spaces to investigate tuberculosis outbreaks in Botswana

**DOI:** 10.1186/s12942-026-00467-5

**Published:** 2026-04-03

**Authors:** Chelsea R. Baker, Ivan Barilar, Leonardo S. de Araujo, Daniel M. Parker, Kimberly Fornace, Patrick K. Moonan, John E. Oeltmann, James L. Tobias, Volodymyr M. Minin, Chawangwa Modongo, Nicola M. Zetola, Stefan Niemann, Sanghyuk S. Shin

**Affiliations:** 1 Sue & Bill Gross School of Nursing, University of California Irvine, 854 Medical Sciences Quad, Irvine, CA 92697 USA; 2 Molecular Mycobacteriology, Forschungszentrum Borstel, Borstel, Germany; 3 Population Health & Disease Prevention, University of California Irvine, CA Irvine, USA; 4 National University of Singapore, Singapore, Singapore; 5 US Centers for Disease Control and Prevention, Atlanta, GA USA; 6 Peraton, 875 Explorer Street, VA Reston, USA; 7 Statistics, University of California Irvine, Irvine, USA; 8 Victus Global Botswana Organisation, Gaborone, Botswana; 9 Augusta University School of Medicine, GA Augusta, USA

**Keywords:** Tuberculosis transmission, Spatial analysis, Activity space, Whole genome sequencing, Geographic heterogeneity, Outbreak

## Abstract

**Objectives:**

Our objective was to identify potential ‘hotspots’ of tuberculosis (TB) transmission by integrating pathogen genomic and geospatial data, including activity spaces where transmission may occur (e.g. community locations such as work, school, or social venues) in addition to residential locations.

**Methods:**

We analyzed geographic data collected during 2012–2016 for the Kopanyo Study, a population-based study of TB transmission in Botswana. We included participants with results from whole genome sequencing conducted on archived samples from the original study. We used a spatial log-Gaussian Cox process model to detect core areas of activity spaces belonging to individuals in TB outbreaks (genotypic groups with ≤ 5 single-nucleotide polymorphisms), which we compared to ungrouped participants (those not in a genotypic group of any size).

**Results:**

We analyzed data for 636 participants, including 70 participants belonging to six outbreak groups with a combined total of 293 locations, and 566 ungrouped participants with a combined total of 2289 locations. Core areas (‘hotspots’) for each outbreak group were geographically distinct, and we found evidence of localized transmission in four of six outbreaks. Hotspots had different geographic characteristics when analyzing activity spaces compared to residential locations alone.

**Conclusions:**

The incorporation of activity spaces in geospatial modeling of genomic data may improve the characterization of potential transmission hotspots.

**Supplementary Information:**

The online version contains supplementary material available at 10.1186/s12942-026-00467-5.

## Background

Tuberculosis (TB) remains a leading cause of death from infectious illness, despite being a preventable and curable disease [1]. In 2023, over 10 million people became sick with TB, and 1.25 million died [1]. Progress toward elimination has been slow and many targets set by the World Health Organization (WHO) remain unmet [1]. New strategies and tools for prevention are urgently needed [1]. In high-burden settings, where a substantial portion of incidence is due to recent infection, interventions to stop ongoing transmission are especially important [1–4].

A promising tool is the integration of geospatial and pathogen genomic data. Whole genome sequencing (WGS) can help identify closely related *M. tuberculosis* isolates and reconstruct likely transmission chains [5]. Geospatial and genomic data can be combined to identify potential areas of sustained transmission and investigate geographic characteristics of outbreaks [6, 7]. This may help identify areas for targeted public health interventions to interrupt ongoing transmission [2–6, 8, 9]. Geographically targeted interventions have shown promise as an effective and cost-efficient strategy for reducing TB incidence in high-burden, low-resource settings [3, 10].

However, this approach has largely been implemented using geographic data for residential locations while excluding other important places in the community where transmission may occur [11, 12]. An alternative approach is to analyze ‘activity spaces,’ which include home as well as other places routinely occupied during daily life, such as workplaces, markets, places of worship, or other social gathering sites [13–15]. This could help improve the characterization of geographic areas potentially involved in TB transmission [14, 16].

We previously conducted a descriptive geospatial analysis using residential locations and WGS data from a population-based study of TB transmission in Botswana and found evidence of geographically distinct outbreaks [17]. The objectives of the current analysis were to use spatial statistical modeling to (1) refine the geographic characterization of outbreaks by incorporating activity spaces, and (2) detect areas with the greatest geographic concentration of activity spaces associated with each outbreak, which could represent ‘hotspots’ of transmission.

## Methods

### Study design and setting

We analyzed data collected during 2012–2016 for the Kopanyo Study, a population-based study of TB transmission in Botswana, a country in Southern Africa with a high burden of TB and TB/HIV co-infection [1, 4, 18]. Participants were recruited at local health clinics in rural and urban health districts [4, 18]. The current analysis focuses on the capital city of Gaborone, which had a population of 354,380 and TB incidence of 440–470 cases/100,000 persons during the five years before the Kopanyo Study [4, 18].

Study participants included men and women of all ages with TB disease who were sequentially enrolled by date of diagnosis [4, 18]. Those who had already received treatment for > 14 days, prisoners, and patients who declined to participate were excluded [4, 18]. At least 1 sputum sample was collected from each participant for bacterial culture [4, 18]. Clinical and demographic data were collected through in-person interviews and medical record review [4, 18].

Activity space data were collected by asking participants about home and social gathering locations (e.g. workplaces, schools, markets, places of worship, alcohol venues etc.) frequented during participants’ potential infectious period (up to 12 months prior to treatment initiation) [4, 18]. All non-residential locations were assigned a unique ID code, to help keep track of locations named by multiple participants and differentiate place names with multiple potential sites (such as supermarket chains with multiple locations). This was done using either street addresses or commonly-known landmarks to help identify the correct location. Participants were specifically asked about common types of places including shopping venues, work, school, places of worship, and alcohol-related venues, though any other types of location reported by participants were recorded as well. Geographic coordinates for latitude and longitude (using WGS 84 projection with 1.1-m precision) were obtained using global positioning system (GPS) devices during site visits, or by geocoding addresses using a reference layer created by manually relocating addresses in satellite imagery using Google Maps, OpenStreetMap, and ArcGIS [4, 18].

### WGS

Whole genome sequencing was conducted on samples archived from the original study with sufficient quantities of DNA (> 0.05 ng/µL) for analysis. Closely related *M. tuberculosis* isolates were identified bioinformatically using a single linkage clustering algorithm. We considered clusters of isolates with ≤ 5 single-nucleotide polymorphisms (SNPs) to indicate recent transmission groups, and groups with ≥ 10 persons were considered outbreaks. Further details of this procedure are outlined in a separate analysis [17]. We chose a cutoff of 5 SNPs based on the threshold associated with epidemiologically linked cases in existing literature [5, 19, 20]. We also identified alternate genotypic cluster groups using a threshold of 12 SNPs, which may be considered an upper limit for transmission [5, 19].

### Spatial analysis of activity space

Participants eligible for the current analysis included those with WGS data, GPS coordinates, and sociodemographic data for age, sex, income, and HIV status. We focused on outbreak groups with at least 10 activity space locations (collectively among all their participants) within greater Gaborone, an area of approximately 27 km x 24 km including the capital city and its surrounding suburbs. Participants were included in analysis if they belonged to one of these groups, and had at least one activity space location within greater Gaborone themselves. Groups with fewer than 10 locations available for analysis were excluded due to difficulty statistically analyzing and detecting robust spatial patterns for very small groups. We also included ungrouped participants (not in a genotypic group of any size) for comparison, in order to characterize activity spaces of people not likely involved in recent transmission and help account for baseline use of space. A very small jitter was introduced to all coordinates (roughly on the scale of different areas of the same building, ranging from approximately < 1 to 10 m), in order to avoid duplicate points as required for our chosen modeling approach.

We conducted a preliminary analysis comparing the geographic distribution of participants with WGS data to all participants in the Kopanyo Study to rule out geographic sampling bias. We estimated the geographic median center (a centralized point that minimizes the distance to all other points), and directional distribution (which calculates the standard deviation of points along the X and Y axes) for both sets of participants and found nearly identical results, indicating that participants with WGS data were geographically representative of the larger study sample. This was performed using ArcGIS [21].

### Model description

We used a spatial log-Gaussian Cox process (LGCP) to model the spatial intensity (average number of points per unit area) of activity spaces of participants belonging to each outbreak group (‘cases’) and ungrouped participants (‘controls’). LGCPs are a flexible class of models for spatial point processes where spatial intensity may vary across the study region [22]. A spatial random effect can be incorporated to account for spatial correlation in the data and identify spatial patterns not explained by other variables [23, 24]. This offers a model-based approach for estimating utilization distributions (UDs), which are probability density functions that can be mapped to highlight areas with increased geographic concentrations of points (e.g. activity space locations) to help characterize use of space [16, 25]. To adapt the modeling framework to an activity space context where each individual may be associated with multiple point locations, observations can be treated as cumulative ‘encounters’ over a specified time period [25]. We considered each point to represent an encounter corresponding to a potential location of TB exposure, and estimated intensity surfaces for cumulative encounters over the entire study period.

LGCPs fit well in a Bayesian hierarchical modeling framework, and various tools can be used for this approach [23, 24]. We used integrated nested Laplace approximation (INLA), a flexible and computationally efficient method for approximate Bayesian inference for latent Gaussian models, which include LGCPs [23, 24, 26]. We implemented this using the R-INLA package [27]. We modeled the spatial random effect as a Gaussian random field (GRF) with Matérn covariance [23, 26]. We used the stochastic partial differential equation (SPDE) approach in R-INLA to approximate the GRF [23, 26]. We specified the SPDE model using penalized complexity priors that were vaguely informative about the underlying spatial process (prior probability of 0.05 that the ranges of the fields were less than 0.5 km and prior probability of 0.05 that the standard deviation was greater than 10).

Under the LGCP framework, we used a joint modeling approach to incorporate a shared spatial term (obtained by jointly estimating the intensity for both cases and controls), as well as a unique spatial term estimated for cases in each group [28, 29]. Using this approach, posterior mean estimates of the spatial random effect for cases represent variation in intensity not accounted for by the spatial distribution of controls [28]. We did this to help identify areas with relatively high concentrations of activity spaces associated with individual outbreak groups, while attempting to account for baseline use of space (e.g. locations frequented more often by people in general). Areas with relatively high density of activity spaces frequented by people in the same outbreak group could potentially represent areas associated with an increased risk of recent transmission. We fit a version of the model that included just the shared spatial term (model 0), and a version of the model that included the shared spatial term as well as unique spatial terms estimated for each outbreak group individually (model 1). We modeled the log-spatial intensity at location x (log(λ(x)) as a sum of fixed effects (an intercept, α) and spatial random effects (S(x)) [28]. The log intensity for controls was log(λ₀(x)) = α₀+ S₀(x) in both models. The log intensity for cases in each of the six genotypic groups (i = 1, 2, 3, 4, 5, 6) was log(λ_i_(x)) = α_i_+ S₀(x) in model 0, and log(λ_i_(x)) = α_i_+ S₀(x) + S_i_(x) in model 1.

We also conducted a sensitivity analysis using subsets of the data with 70 and 140 randomly selected ungrouped participants as controls to examine whether spatial patterns were sensitive to size of the control group. In addition, we conducted a sensitivity analysis using an alternate jitter of 5 m applied to all points to assess whether spatial patterns were sensitive to jittering. We also included a sensitivity analysis of genotypic groups identified using the alternate 12-SNP cutoff to examine differences in spatial patterns using different SNP thresholds.

We projected posterior mean estimates of the spatial effect (i.e. the effect of spatial location on the intensity of activity spaces) for each outbreak group onto maps of the study area to identify areas of increased or decreased (different than zero) values not explained by the spatial distribution of controls [28]. Estimated values (displayed on the internal linear predictor scale) represent the contribution of the spatial random effect to the response (spatial intensity) after accounting for other fixed and random effects in the model. We then projected posterior mean estimates of predicted spatial intensity values (fitted values of the response), in order to visualize predicted spatial patterns of activity spaces for each group [26, 29].

In addition, we calculated and mapped exceedance probabilities to identify high-confidence areas where the estimated spatial effect for each group had a high probability (0.95) of being greater than the baseline effect estimated from the spatial distribution of the control group [28]. We also generated exceedance maps for spatial intensity, identifying high-confidence areas where posterior mean intensity values had a high probability (0.95) of being in the upper 10% of estimates for that group, representing ‘core areas’ or ‘hotspots’ of that group’s collective activity space [25]. For comparison, we also generated exceedance maps for spatial effects and spatial intensity based on residential locations alone, using the same threshold values as above.

Map visualization was performed using the R packages raster, terra, sf, ggplot2, ggspatial, and leaflet.

## Results

### Participants

A total of 1426 participants had WGS data available, of which 1425 had GPS coordinates available for at least one activity space location (home or social gathering place). Participants with and without WGS data had similar sociodemographic characteristics in terms of age, sex, HIV status, and income. Over half of all genotypic groups (*n* = 107, 55%) had only two cases, and another 30% (*n* = 58) had 5 or fewer cases. Eight genotypic groups had 10 participants or more and were considered outbreaks. After excluding participants with no individual locations within the study area, six of the eight outbreak groups had at least 10 activity spaces (collectively among all their participants) in greater Gaborone, although this geographic exclusion resulted in two of the groups retaining fewer than 10 individual participants. A total of 636 participants met criteria for the current analysis, including 70 participants belonging to six outbreak groups with a combined total of 293 locations, and 566 ungrouped participants with a combined total of 2289 locations. The number of activity spaces at the same geographic coordinate location before spatial jittering ranged from one to 48, with over half all locations (*n* = 1193) having unique coordinates before jittering (Supplementary Fig. 1).

Each participant had between one and 10 activity space locations, and the median number of locations (*n* = 4) was the same for both grouped and ungrouped participants. The median number of activity spaces was also the same (*n* = 4) by gender and HIV status, though was slightly lower for participants with no income (*n* = 3) than participants with any income (*n* = 4), which could reflect an increased number of activity spaces among participants who were employed. Among participants with two or more mapped locations, the maximum distance between activity spaces ranged from < 0.5 km to 21.2 km (median 6.2 km) for ungrouped participants and < 0.5 km to 21.7 km (median 4.3 km) for participants in outbreak groups (supplementary Fig. 2). While the range of overall maximum distances traveled was similar, the median of maximum distances may have been different between grouped and ungrouped participants (Wilcoxon rank-sum test p-value 0.06).

Among genotypically ungrouped participants, the median age was 35 years (IQR: 28–42), just over half were male, about one quarter reported no income, and nearly 65% were diagnosed with TB-HIV coinfection (Table 1). Among participants in the six genotypic groups, median age ranged from 30 years (Group A) to 39 years (Group G). Participants in Group G were exclusively male, while Group C alone was majority female (75%). Group D had the highest proportion of participants diagnosed with TB-HIV coinfection (9 of 11; 91%). The percentage of participants reporting no income ranged from 18% in Group D to 58% in Groups C and E (Table 1).

**Table 1:** Characteristics of study participants (*N* = 636) by outbreak group (genotypic group ≤ 5 SNP), Gaborone, Botswana, 2012–2016

	A (*N* = 22)	C (*N* = 12)	D (*N* = 11)	E (*N* = 12)	G (*N* = 9)	H (*N* = 4)	Ungrouped (*N* = 566)
Total locations	81	45	53	54	44	16	2289
Gender							
Female	11 (50.0%)	9 (75.0%)	5 (45.5%)	3 (25.0%)	0 (0%)	2 (50.0%)	264 (46.6%)
Male	11 (50.0%)	3 (25.0%)	6 (54.5%)	9 (75.0%)	9 (100%)	2 (50.0%)	302 (53.4%)
Age							
Median [Q1,Q3]	29 [24, 37]	31 [29, 36]	33 [31, 42]	35 [29, 40]	39 [35, 42]	24 [20, 38]	35 [28, 42]
HIV Status							
Neg	10 (45.5%)	5 (41.7%)	1 (9.1%)	6 (50.0%)	4 (44.4%)	3 (75.0%)	203 (35.9%)
Pos	12 (54.5%)	7 (58.3%)	10 (90.9%)	6 (50.0%)	5 (55.6%)	1 (25.0%)	363 (64.1%)
Income							
Any	16 (72.7%)	5 (41.7%)	9 (81.8%)	5 (41.7%)	7 (77.8%)	2 (50.0%)	417 (73.7%)
None	6 (27.3%)	7 (58.3%)	2 (18.2%)	7 (58.3%)	2 (22.2%)	2 (50.0%)	149 (26.3%)

Using the alternate 12 SNP threshold, a total of 542 participants met inclusion criteria, including 158 participants belonging to nine outbreak groups with a combined total of 661 locations, and 384 ungrouped controls with a combined total of 1551 locations (Supplementary Table 2).

### Estimated spatial effects

Model 1 (shared and group-specific spatial terms) had a lower DIC (−12956.58) than model 0 (shared spatial terms only, DIC − 12853.84), supporting the presence of spatial variation among genotypic groups not accounted for by the spatial distribution of activity spaces of controls [28].

**Fig. 1:**
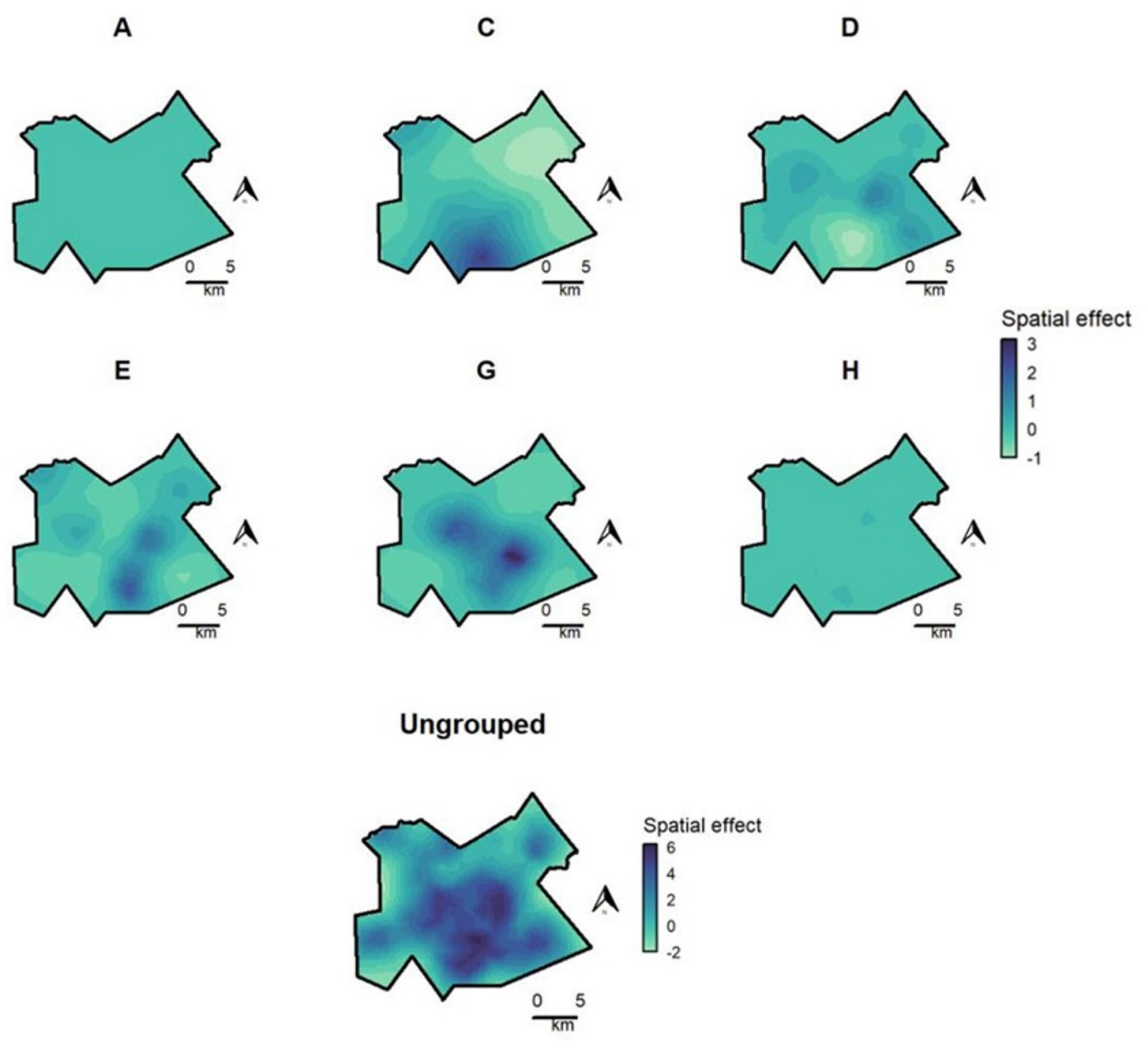
Posterior mean estimates of spatial random effects for outbreak groups (**A-H**) and controls (ungrouped participants), Gaborone, Botswana, 2012–2016. Values are shown on the internal linear predictor scale and represent the contribution of the spatial random effect to the response, after accounting for other fixed and random effects in the model. Darker colors correspond to increased spatial effect estimates. Values are displayed on the same color scale for all outbreak groups, though on a separate color scale for controls due to difference in sample size

Maps of estimated spatial effects revealed distinct spatial patterns among outbreak groups (Fig. 1). The spatial effect for groups A and H was relatively small and showed little spatial variation compared to the baseline represented by the control group. Group C had a notable area of increased values in the central southern part of the study area. Groups D and G had several areas of increased values broadly following a northwest to southeast track, while group E had several areas of increased values following a north-south configuration.

Results of the sensitivity analysis using subsets of 70 and 140 randomly selected controls found very similar results in terms of the spatial patterns and magnitude of estimated spatial effect by group (Supplementary Fig. 2 and Supplementary Fig. 4). Similarly, results were robust to the alternate jitter, with spatial patterns nearly identical to the original analysis (Supplementary Fig. 8). In the 12-SNP sensitivity analysis, there were several overlapping areas of increased spatial effect among the alternate and original groups, particularly for groups B12, E12, H12, and I12 (Supplementary Fig. 10). Spatial patterns for alternate groups A12 and D12 were less distinct, and alternate groups F12, G12, and J12 were similar to ungrouped controls (Supplementary Fig. 10). The spatial distribution of ungrouped participants was very similar to the original analysis (Supplementary Fig. 10).

### Predicted spatial intensity

**Fig. 2:**
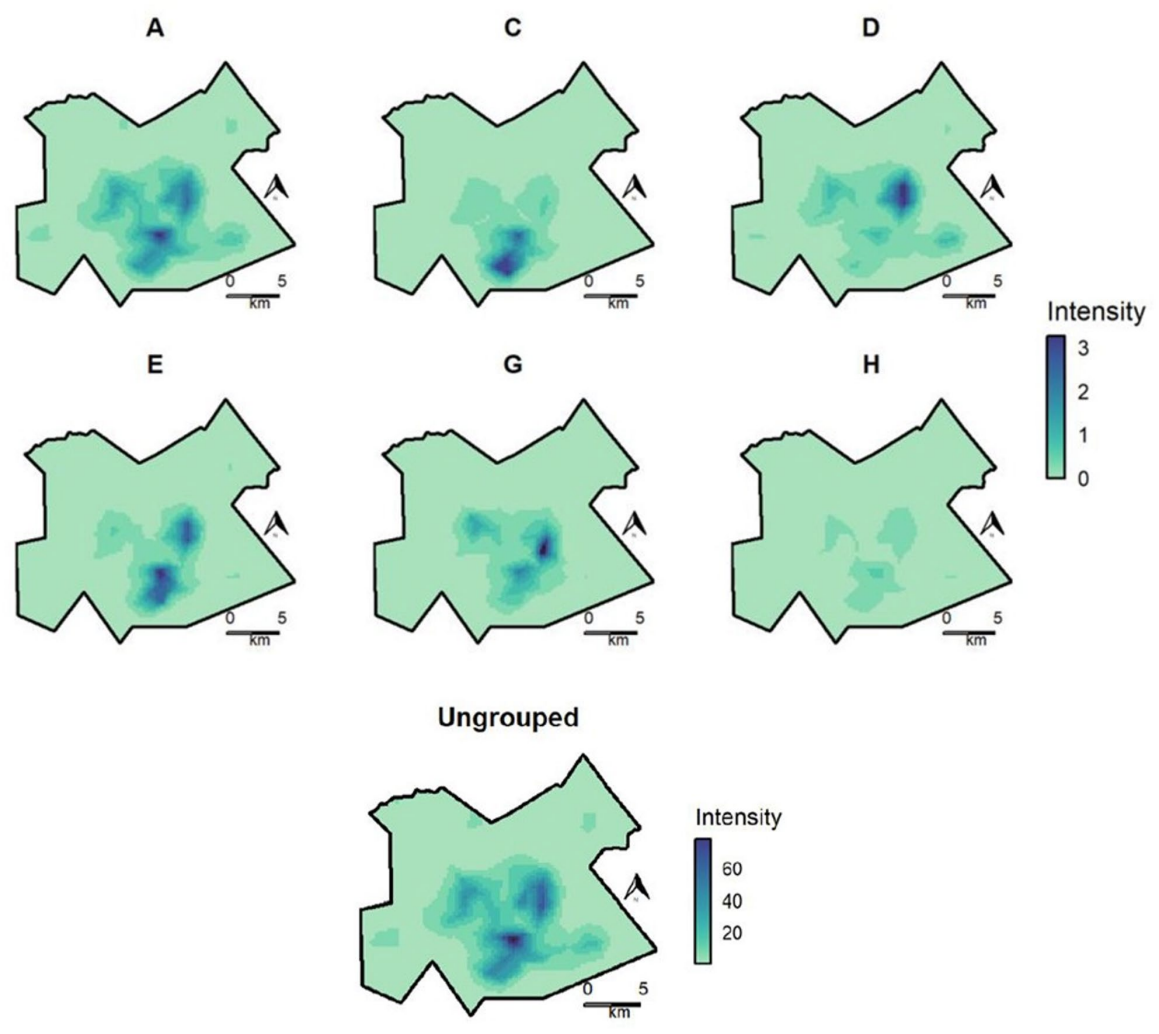
Predicted mean spatial intensity, outbreak groups (**A-H**) and controls (ungrouped participants), Gaborone, Botswana, 2012–2016. Values represent posterior mean values for predicted numbers of activity spaces per unit area (approximately 0.25 × 0.25 km). Intensity values are displayed on the same color scale for all outbreak groups, though on a separate color scale for controls due to difference in sample size

Maps of predicted mean spatial intensity of activity spaces displayed distinct spatial patterns among groups (Fig. 2). For groups A and H, areas of increased intensity followed an overall pattern similar to the ungrouped controls, with areas of highest intensity toward the center of the study area. Group C had a distinct area of high intensity in the central southern part of the study area. Group D had a notable area of increased intensity in the central east part of the map. Areas of highest intensity for group E were in the central and south east, and for group G in the central east.

Results of the sensitivity analysis using subsets of 70 and 140 randomly selected controls found very similar results for predicted spatial intensity by outbreak group (Supplementary Fig. 3 and Supplementary Fig. 5). Results using the alternate jitter were nearly identical to the original analysis (Supplementary Fig. 9). In the 12-SNP sensitivity analysis, there were several overlapping areas where the highest predicted intensity for alternate groups were similar to groups in the original analysis, particularly for groups B12, E12, H12, and I12 (Supplementary Fig. 11).

### Exceedance maps

**Fig. 3:**
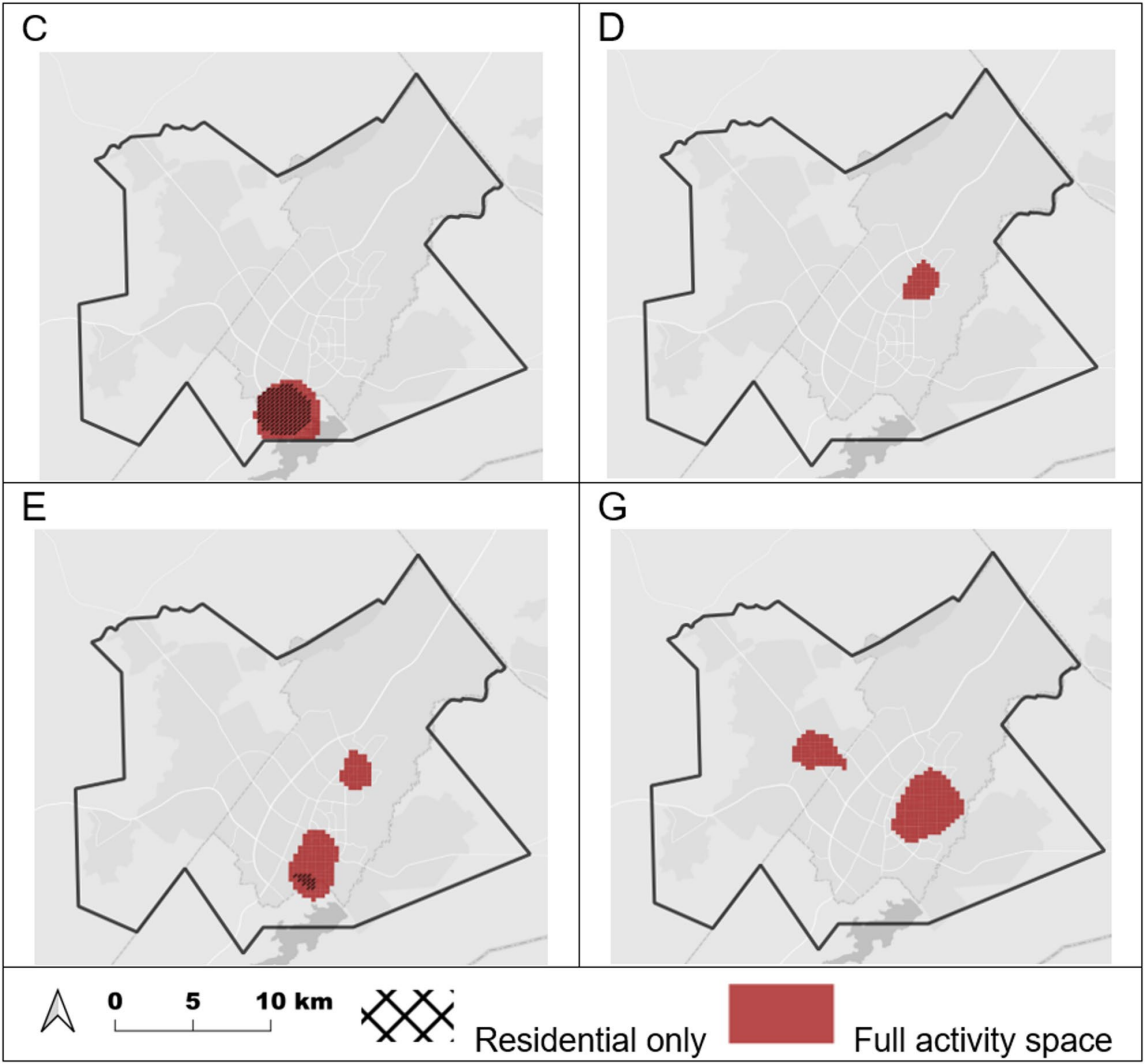
Estimated spatial effect exceedance maps, full activity space and residential only, Gaborone, Botswana, 2012–2016. Shaded areas have a high probability (0.95) of having a value greater than 0 (departure above baseline of control group)

Exceedance maps for estimated spatial effects based on full activity space analysis displayed significant areas of increased estimates for groups C, D, E, and G (Fig. 3). Groups A and H did not have areas meeting the specified threshold. Exceedance maps for estimated spatial effects based on residential location alone displayed significant areas of increased estimates for groups C and E only. Group C had a similar exceedance area in both analyses, while group E had two relatively large exceedance areas in the activity space analysis, compared to one small area for residential location alone.

**Fig. 4:**
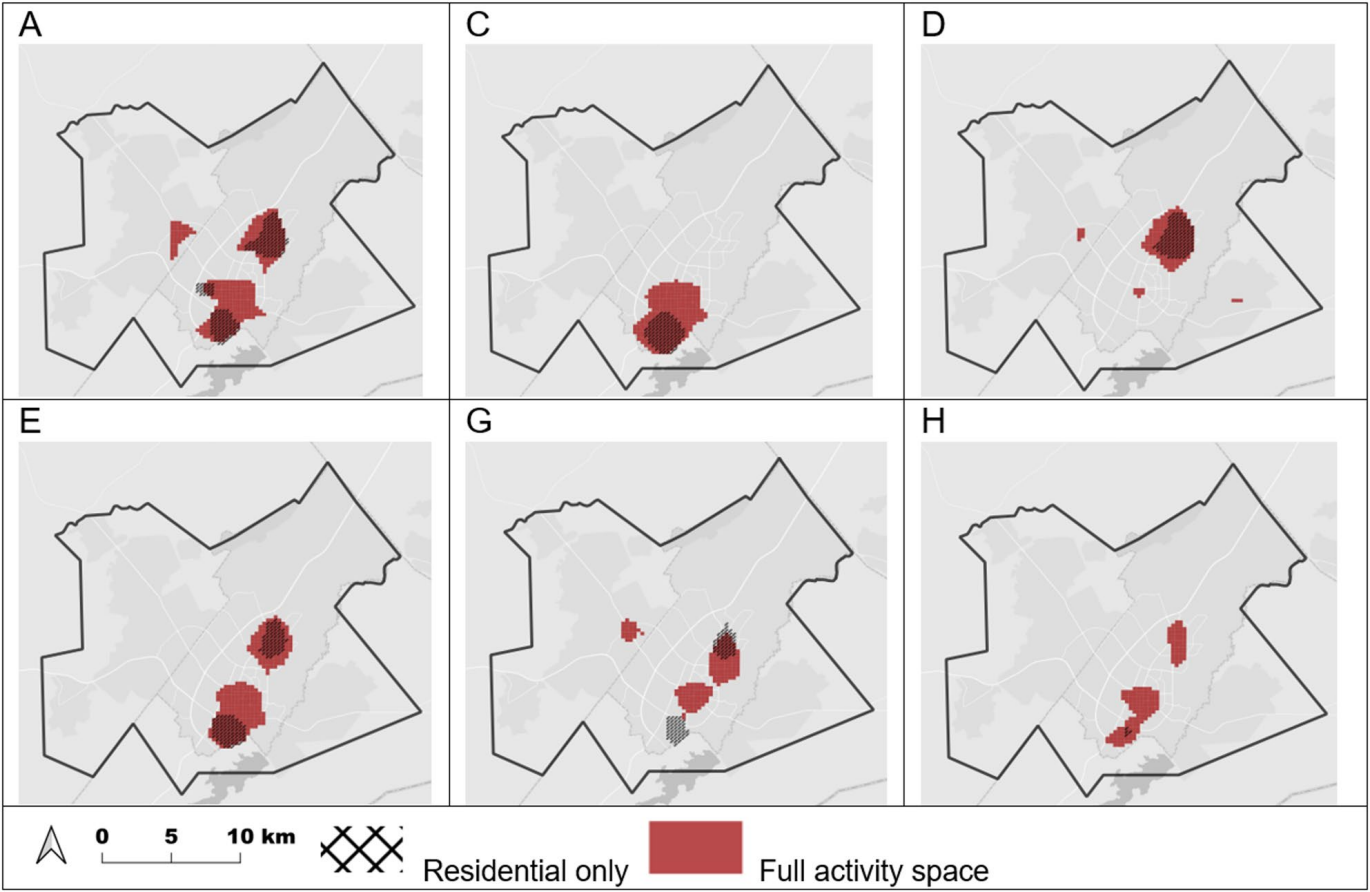
Predicted spatial intensity exceedance maps, full activity space and residential only, Gaborone, Botswana, 2012–2016. Shaded areas represent ‘core areas’ of activity space for each outbreak group

Exceedance maps for predicted spatial intensity values showed geographically distinct core areas of greatest spatial intensity (values in the top 10%) for each outbreak group (Fig. 4). For all groups, core areas based on full activity space analysis were larger than those based on residential locations alone. For groups A, D, G, and H, core areas based on activity space also involved additional areas within the study region.

## Discussion

We detected geographically distinct patterns of activity spaces associated with different TB outbreak groups. Geographic characteristics for groups A (the largest outbreak group) and H (the smallest) were similar to ungrouped controls. For groups C, D, E, and G, we detected areas where the estimated spatial random effect was significantly higher than the baseline spatial distribution of ungrouped controls. Core areas (‘hotspots’) containing the highest spatial concentration of activity spaces for each group were located in different areas, with some being more geographically widespread and others more compact. This could suggest that distinct areas of localized transmission play an important role in some outbreaks. Differences in spatial characteristics among the groups could represent transmission among socially or geographically distinct contact networks. Spatial patterns could also be influenced by the timing of how long a genotype of TB has been circulating in the community.

Results of 12-SNP sensitivity analysis identified alternate genotypic groups with distinct but partially overlapping spatial characteristics compared to the original analysis. Three of the alternate groups had similar spatial patterns to ungrouped controls, while two groups had distinct characteristics on a relatively broad scale, and four groups displayed more unique localized spatial patterns. A threshold of 12 SNPs is less specific to recent transmission [5, 19, 20], and these spatial patterns may reflect larger numbers of individuals not involved in spatially distinct transmission chains. A threshold of 12 SNPs may also reflect transmission over a longer time period [5, 19, 20], which our current activity space data may not account for, as relevant locations could have changed over time.

Overall, grouped participants may have had smaller movement ranges than ungrouped participants. This could potentially result in increased contact and repeated exposures, further increasing the risk of transmission [30]. The total number of activity spaces was similar among grouped and ungrouped participants. There were some individuals who did not have any non-residential locations. This could possibly be due to missing data or locations that were excluded because they were outside the study area being analyzed. These individuals also may have been doing labor close to home or were unemployed. It is also possible that some people were not comfortable sharing their activity space information with the study team. The response of no non-residential activity space is a topic that could be interrogated further in future studies.

We also found differences in areas of core spatial intensity and significant spatial effects that were detected using full activity space analysis compared to residential location alone. In general, areas based on activity space analysis were generally larger and sometimes included additional parts of the study region. This could suggest a notable portion of activity spaces may be located in areas outside participants’ home neighborhoods. An exception is group C, which had similar areas identified using activity space and residential-only analyses. Sociodemographic characteristics such as relatively high unemployment (relatively few work locations) could be a contributing factor. However, our results show that in many cases analysis based on residential location alone may not fully represent the spatial characteristics of potential transmission hotspots.

Our results are in line with studies of TB in the US [15] and South Africa [11] which both noted differences between ‘high-risk’ areas identified using activity spaces compared to residential locations alone. These studies highlighted the impact of activity space analysis on geographic characterization of areas of potential transmission. Our study built on this approach by incorporating genomic data to strengthen the detection of potential recent transmission.

Our results are also in line with a recent study in Peru that combined WGS and spatial data to identify differences in activity spaces of genotypically related and unrelated cases and non-TB controls [16]. Notably, this study drew on methodology from spatial ecology, using UDs to model activity space at the individual and group level [16]. The authors focused mainly on quantifying the size and amount of overlap among participants’ UDs, rather than identifying specific high-risk areas. Our study expanded on these methods by characterizing UDs using a spatial point process model, which allowed us to adjust for baseline use of space, and detect high-confidence areas of interest in the community.

Other recent TB studies have also emphasized the importance of incorporating a spatial control group for detecting epidemiologically relevant spatial patterns [31–33], and demonstrated the use of spatial random effects to detect potential high transmission risk areas [32, 34]. We demonstrated how these elements can be incorporated in a Bayesian hierarchical model for activity space analysis.

In our analysis, we did not explicitly identify activity space locations frequented by multiple participants (‘shared activity spaces’). This could be an alternate approach for studying activity space for TB transmission. Several recent studies have incorporated genomic data and social network representations that link participants to common social gathering locations in the community [35–37]. Further analysis could focus on shared activity spaces among members of the same genotypic group to identify potential sites of transmission in the current study setting.

Spatial analysis for infectious disease transmission involves an inherent assumption that the locations analyzed are important with regard to transmission. Activity space analysis incorporates important locations in the community where TB transmission could occur, and may reduce exposure misclassification and improve the geographic characterization of transmission chains [11, 16]. This has implications for planning targeted interventions. Our approach could be used to identify potential areas to prioritize for public health outreach such as active case finding. However, a major challenge remains the long delay in obtaining actionable genomic data, limiting the current use of these methods for real-time public health action.

A limitation of our study is that we may have included activity space locations that are not relevant to transmission. Another limitation is that we did not examine additional risk factors. Spatial variation is often a proxy for unmeasured social, structural, or environmental variables [26, 38]. The spatial LGCP modeling approach can incorporate spatially referenced covariates [38], however these data were not available for our analysis. We also did not include specific measures of temporality, which is an important element of transmission dynamics. Further analysis could incorporate additional data to examine other variables.

Another limitation of this study is an unknown number of missing cases, activity space locations, and WGS data that could potentially alter geographic characterization of genotypic groups. Although the original study had relatively high enrollment (4,331/5,515 persons diagnosed during the study period), not every person with TB was included, such undetected cases and those diagnosed but not enrolled. In addition, location data obtained through patient interviews is subject to recall bias and underreporting [39]. Other methods such as prospective GPS tracking have been suggested as potential alternatives [16]. However, locations visited during the infectious period prior to study enrollment were of primary interest in this analysis. Further, a study comparing self-report to GPS logger data found that for three quarters of respondents, over 70% of self-reported locations matched with the GPS data [40].

## Conclusion

Integrating genomic and geospatial data is a powerful tool to help understand TB transmission. This approach may be strengthened by analyzing activity spaces rather than residential locations alone, which could improve the geographic characterization of transmission ‘hotspots’. This could help with planning and mobilizing interventions to interrupt ongoing transmission, and could provide a valuable tool for public health officials working to eliminate TB among marginalized communities [6].

## Supplementary Information

Supplementary Material 1

## Data Availability

Data for the geographic locations of study participants is confidential and not publicly available.
