# Supplementary material for: Using genomic epidemiology and geographic activity spaces to investigate tuberculosis outbreaks in Botswana
Source: Int J Health Geogr. 2026 Apr 3;25:30. doi: 10.1186/s12942-026-00467-5 (PMC13173891; doi:10.1186/s12942-026-00467-5)

**Supplementary Figure 1. Histogram for number of activity space locations at the same geographic coordinate location before spatial jittering, Gaborone, Botswana, 2012-2016.** All together there were 1420 unique coordinate points before jittering and 2582 after. The number of activity spaces at the same geographic coordinate location before jittering ranged from 1 to 48.

**
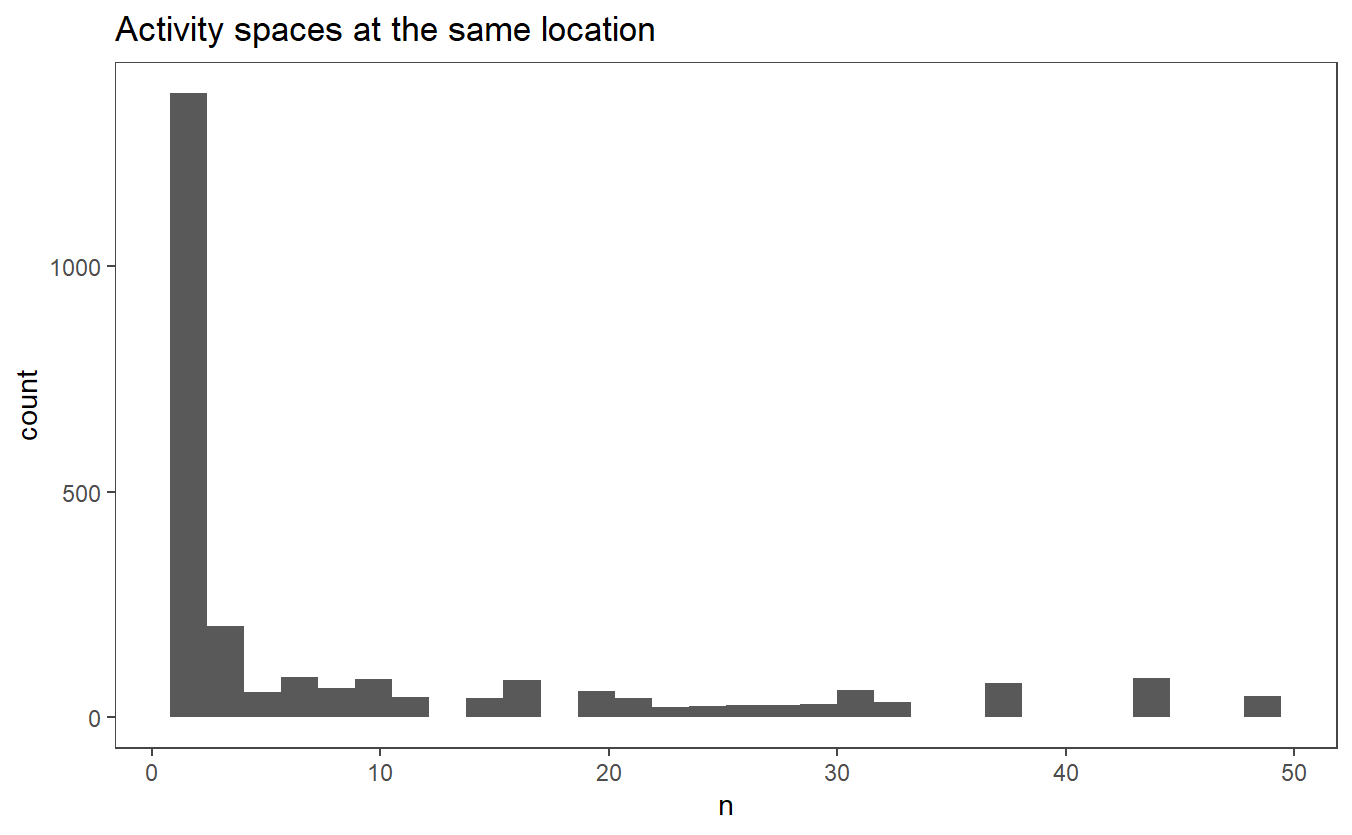
**

**Supplementary Figure 2**. **Maximum distance between any two of a participant's own activity spaces, among participants belonging to outbreak groups (≤5 SNP) and ungrouped participants, Gaborone, Botswana, 2012-2016.**

| 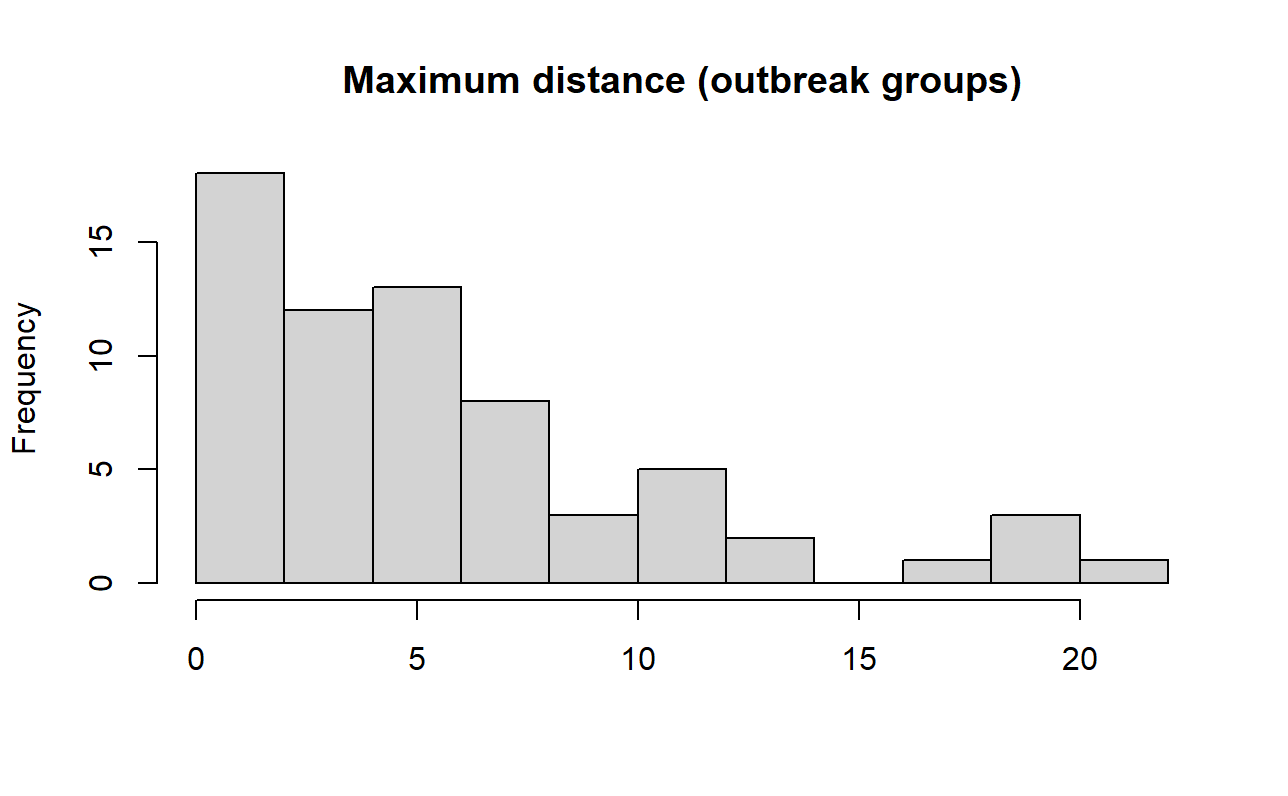 | 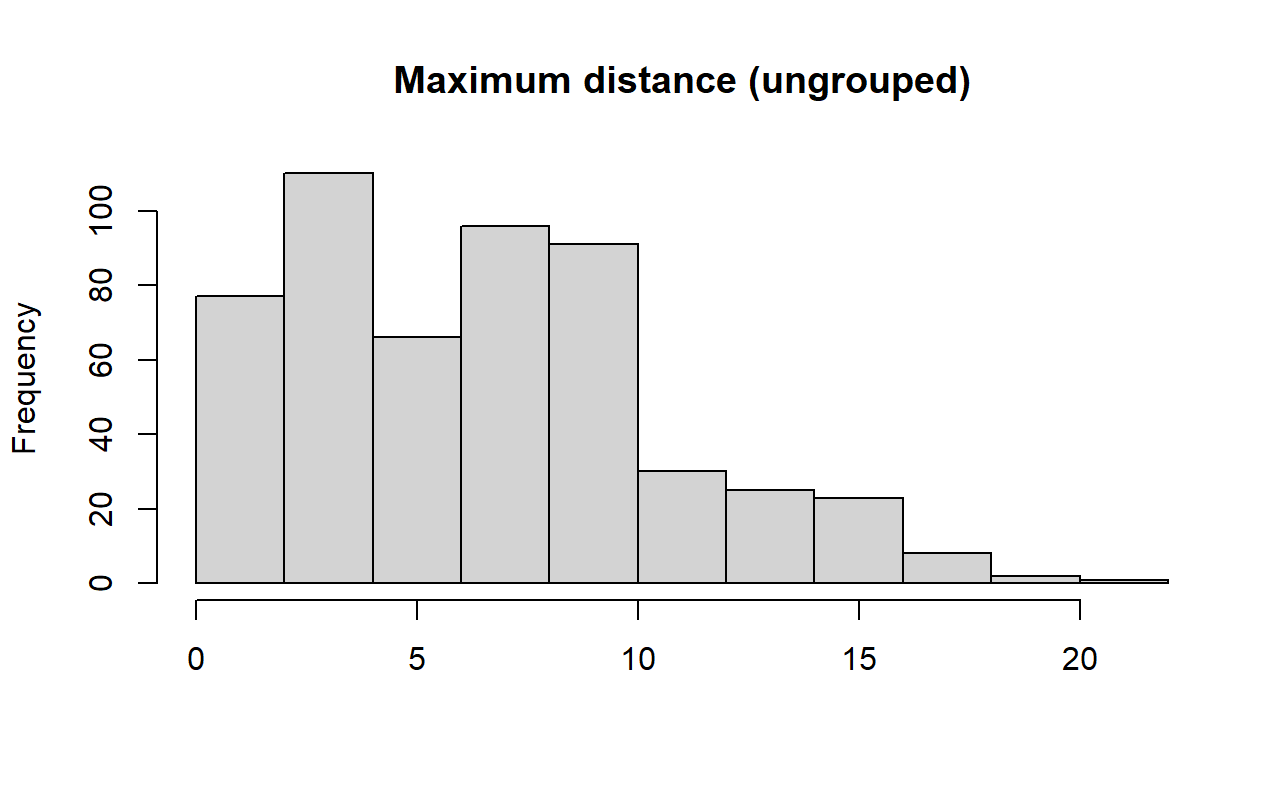 |
| --- | --- |

**Supplementary Table 1.** **Maximum distance between any two of a participant's own activity spaces, by sociodemographic category among participants belonging to outbreak groups (≤5 SNP) and ungrouped participants, Gaborone, Botswana, 2012-2016.**

|  | **Outbreak groups** | **Ungrouped** |
| --- | --- | --- |
| HIV neg | Median 4 (range 1 - 8) | Median 4 (range 1 - 9) |
| HIV pos | Median 4 (range 1 - 10) | Median 4 (range 1 - 9) |
| Income any | Median 4 (range 2 – 10) | Median 4 (range 1 - 9) |
| Income none | Median 3.5 (range 1 – 5) | Median 3 (range 1 - 9) |
| Gender female | Median 4 (range 1 – 6) | Median 4 (range 1 - 9) |
| Gender male | Median 4 (range 1 – 10) | Median 4 (range 1 - 9) |

**Supplementary Table 2. Genotypic groups identified using alternate 12 SNP threshold, Gaborone, Botswana, 2012-2016.**

| Group | A | B | D | E | F | G | H | I | J | Ungrouped |
| --- | --- | --- | --- | --- | --- | --- | --- | --- | --- | --- |
| N participants | 19 | 67 | 14 | 12 | 5 | 11 | 12 | 10 | 8 | 384 |
| N locations | 81 | 261 | 68 | 60 | 19 | 51 | 57 | 35 | 29 | 1551 |

**Supplementary Figure 3. Posterior estimates of the range of the estimated spatial effect for each outbreak group, Gaborone, Botswana, 2012-2016.**


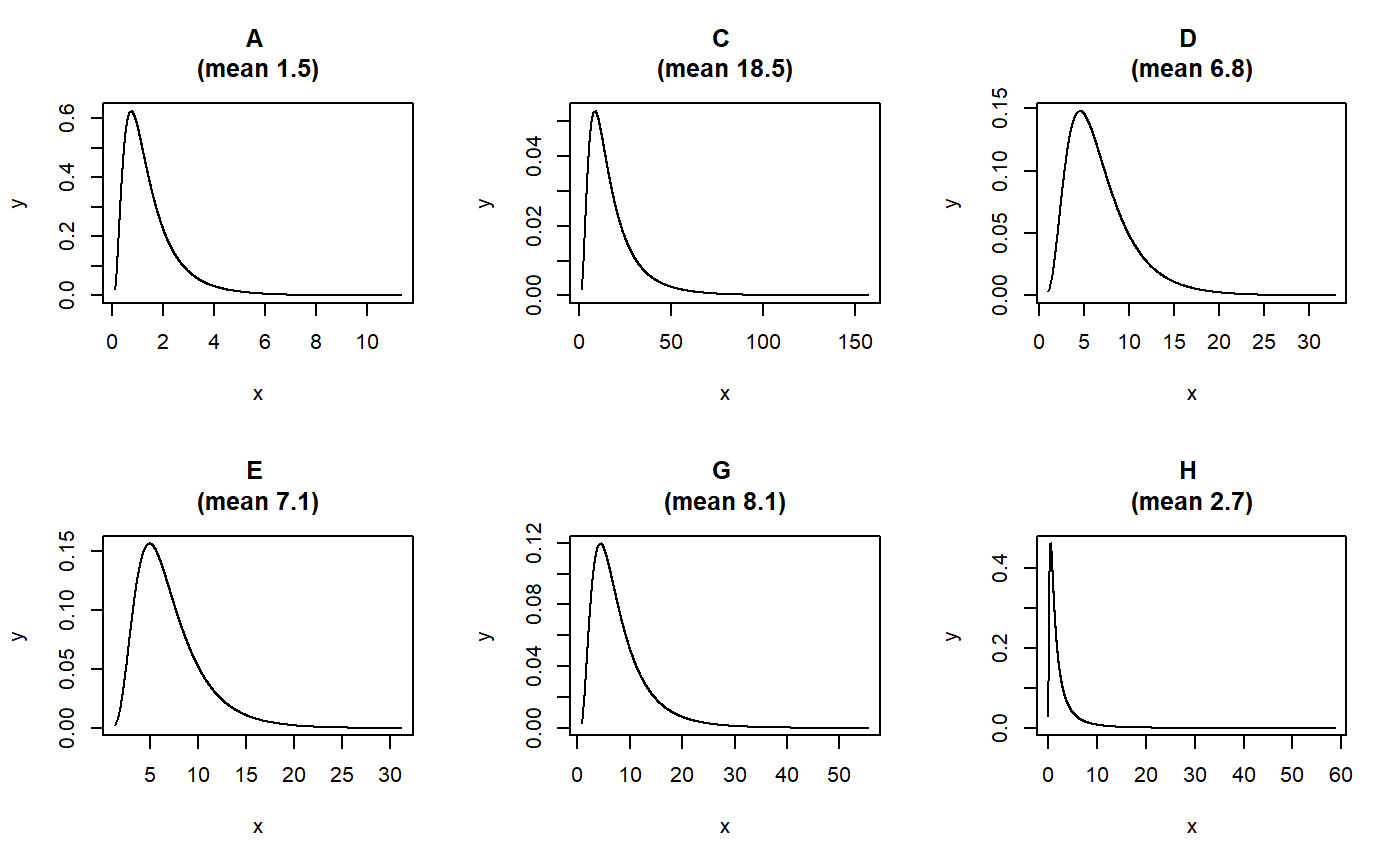


**Supplementary Figure 4.** **Posterior mean estimates of spatial random effect for each outbreak group and subset of 70 randomly selected controls (ungrouped participants) Gaborone, Botswana, 2012-2016.** Darker colored areas indicate places where the estimated spatial effect associated with activity spaces for participants in each group was increased after accounting for controls. Values are displayed on the same color scale for all outbreak groups, though on a separate color scale for controls due to difference in sample size.


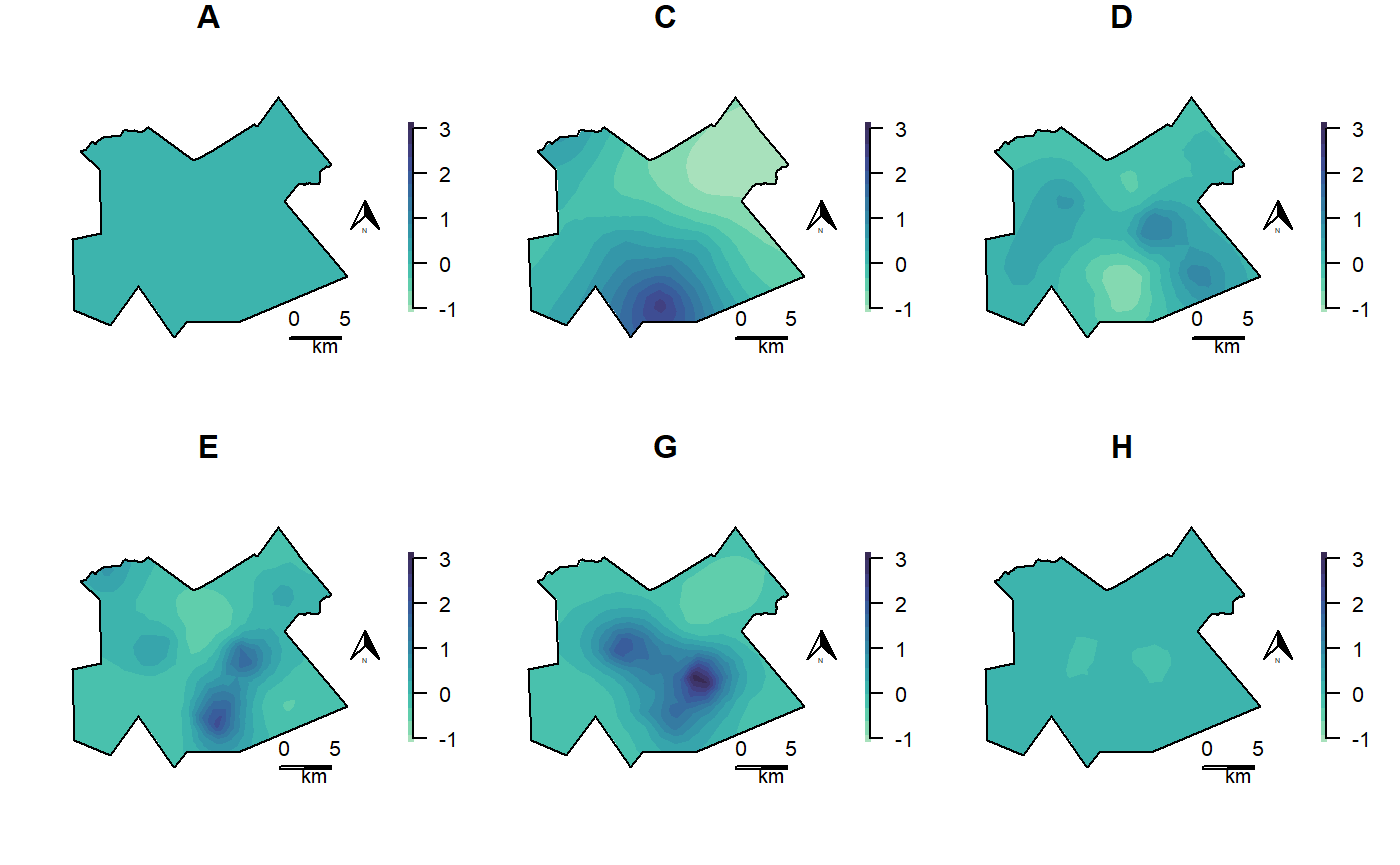


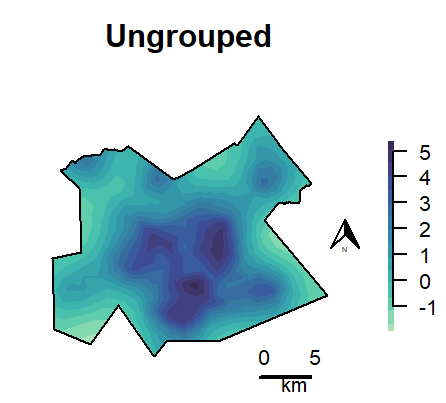


**Supplementary Figure 5.** **Predicted mean spatial intensity of activity spaces for participants in each outbreak group and random subset of 70 ungrouped participants, Gaborone, Botswana, 2012-2016.** Values are displayed on the same color scale for all outbreak groups, though on a separate color scale for controls due to difference in sample size.


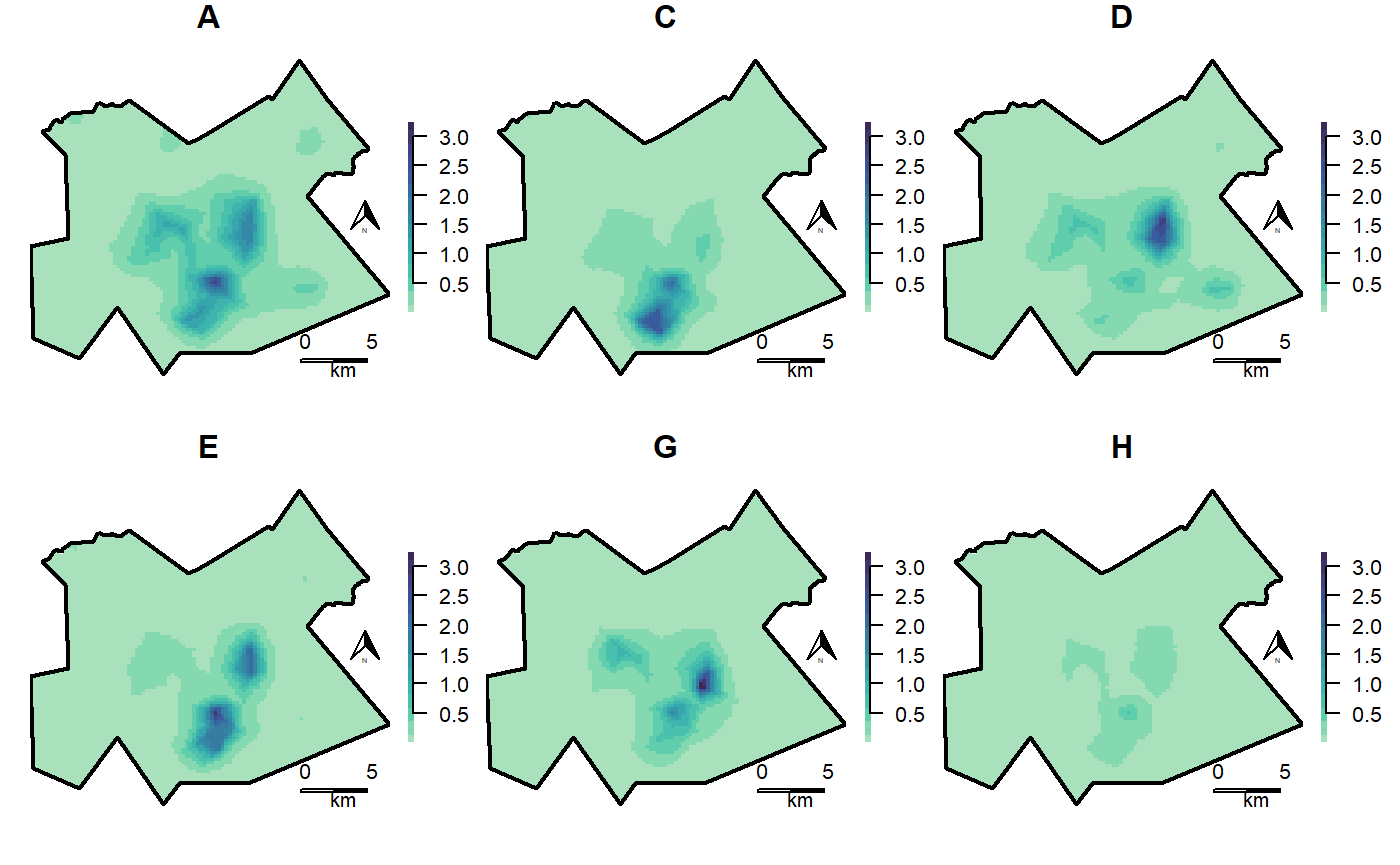


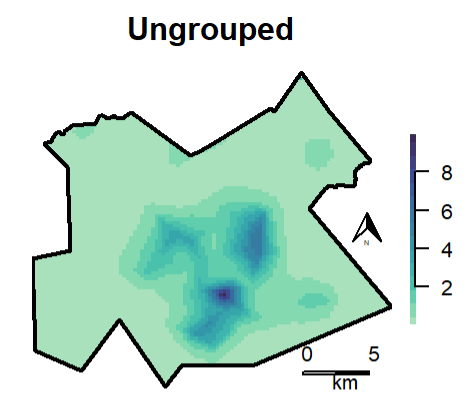


**Supplementary Figure 6.** **Posterior mean estimates of spatial random effect for each outbreak group and subset of 140 randomly selected controls (ungrouped participants) Gaborone, Botswana, 2012-2016.** Darker colored areas indicate places where the estimated spatial effect associated with activity spaces for participants in each group was increased after accounting for controls. Values are displayed on the same color scale for all outbreak groups, though on a separate color scale for controls due to difference in sample size.


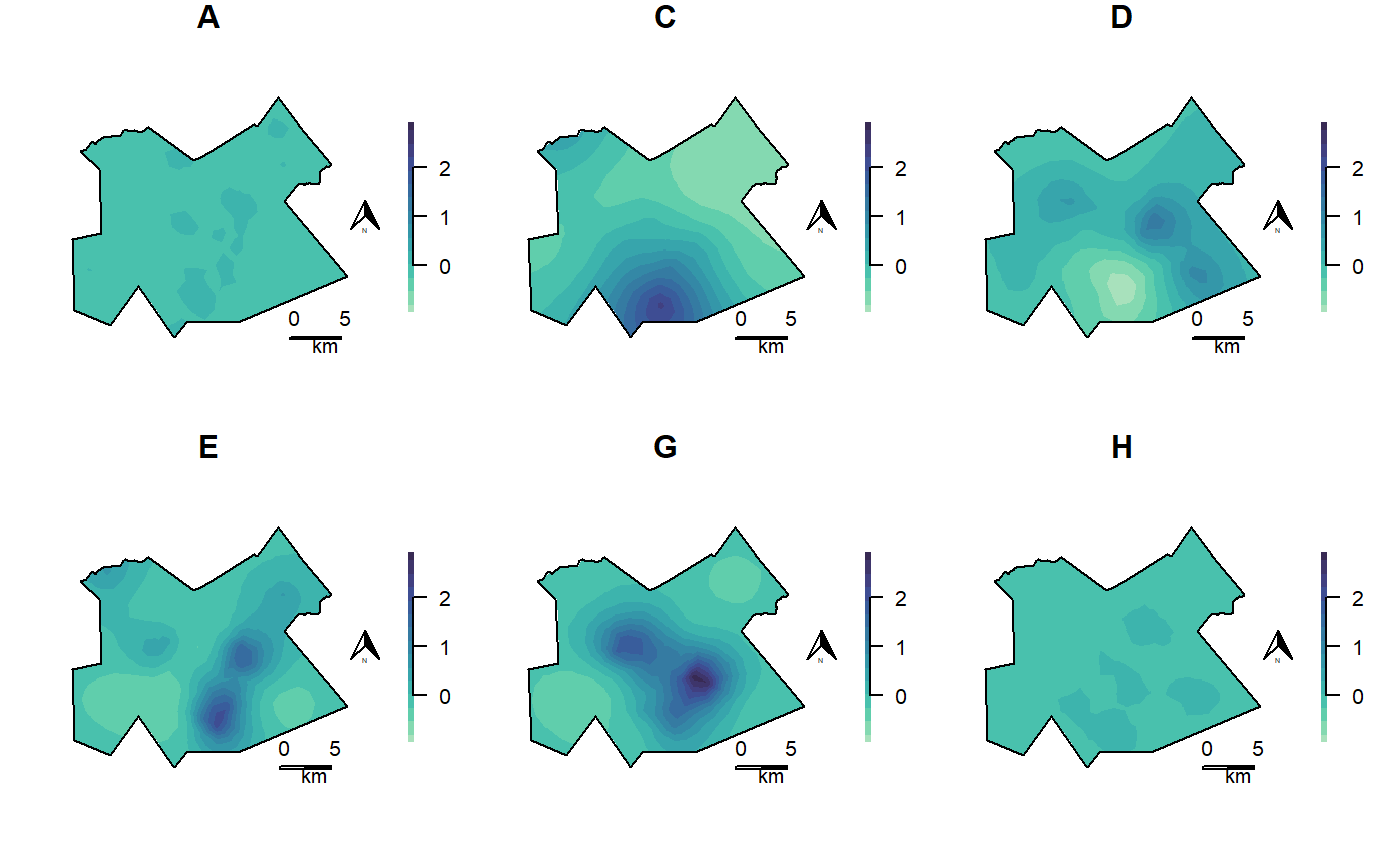


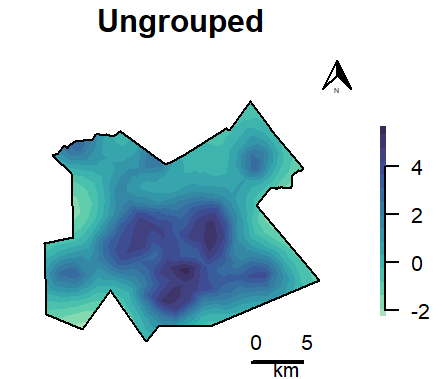


**Supplementary Figure 7.** **Predicted mean spatial intensity of activity spaces for participants in each outbreak group and random subset of 140 ungrouped participants, Gaborone, Botswana, 2012-2016.** Values are displayed on the same color scale for all outbreak groups, though on a separate color scale for controls due to difference in sample size.


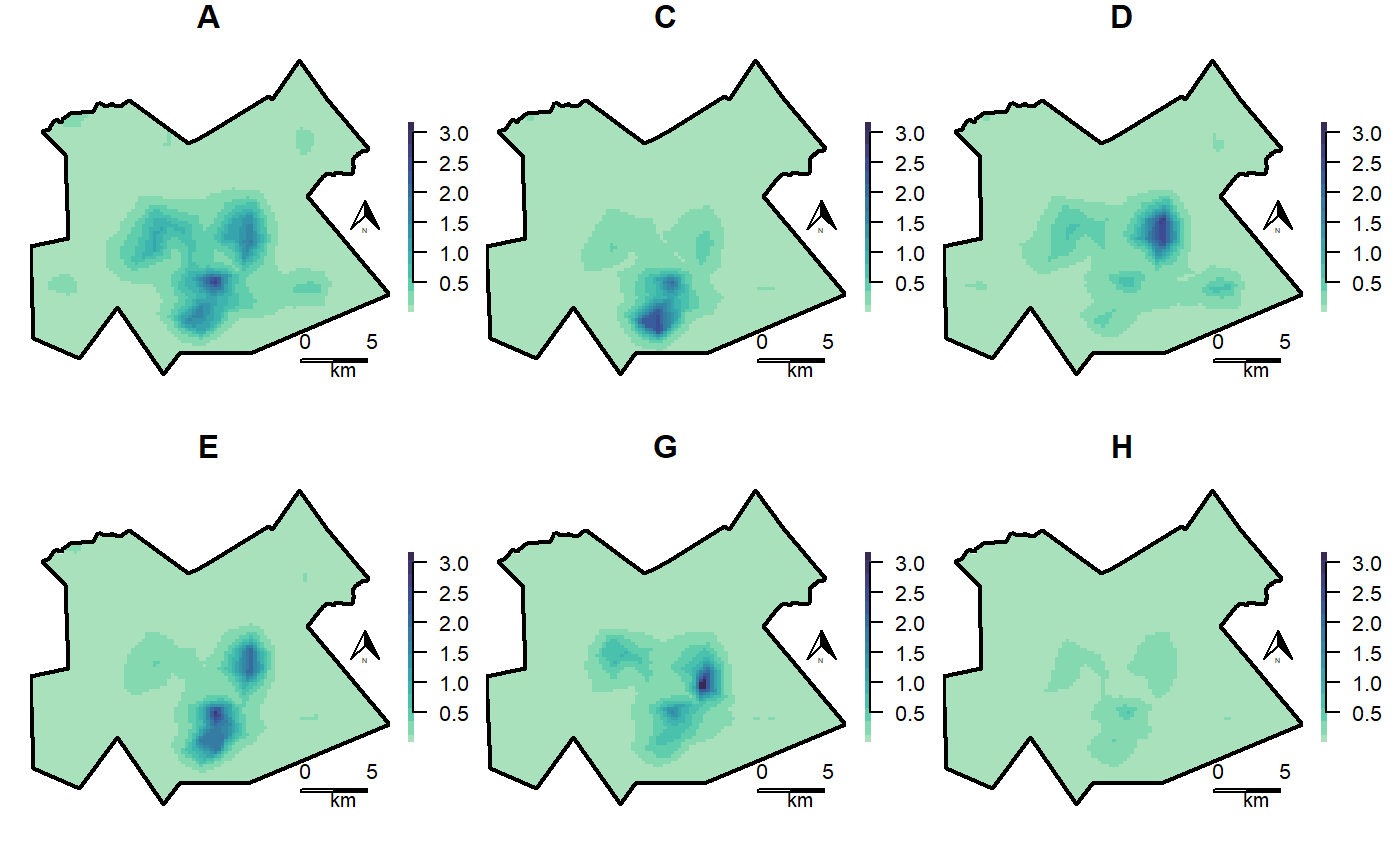


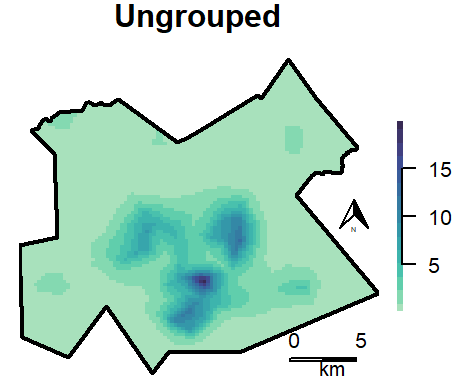


**Supplementary Figure 8. Posterior mean estimates of spatial random effect for each outbreak group and controls (ungrouped participants) using an alternate spatial jitter, Gaborone, Botswana, 2012-2016.** Darker colored areas indicate places where the estimated spatial effect associated with activity spaces for participants in each group was increased after accounting for controls. Values are displayed on the same color scale for all outbreak groups, though on a separate color scale for ungrouped controls due to difference in sample size.

**
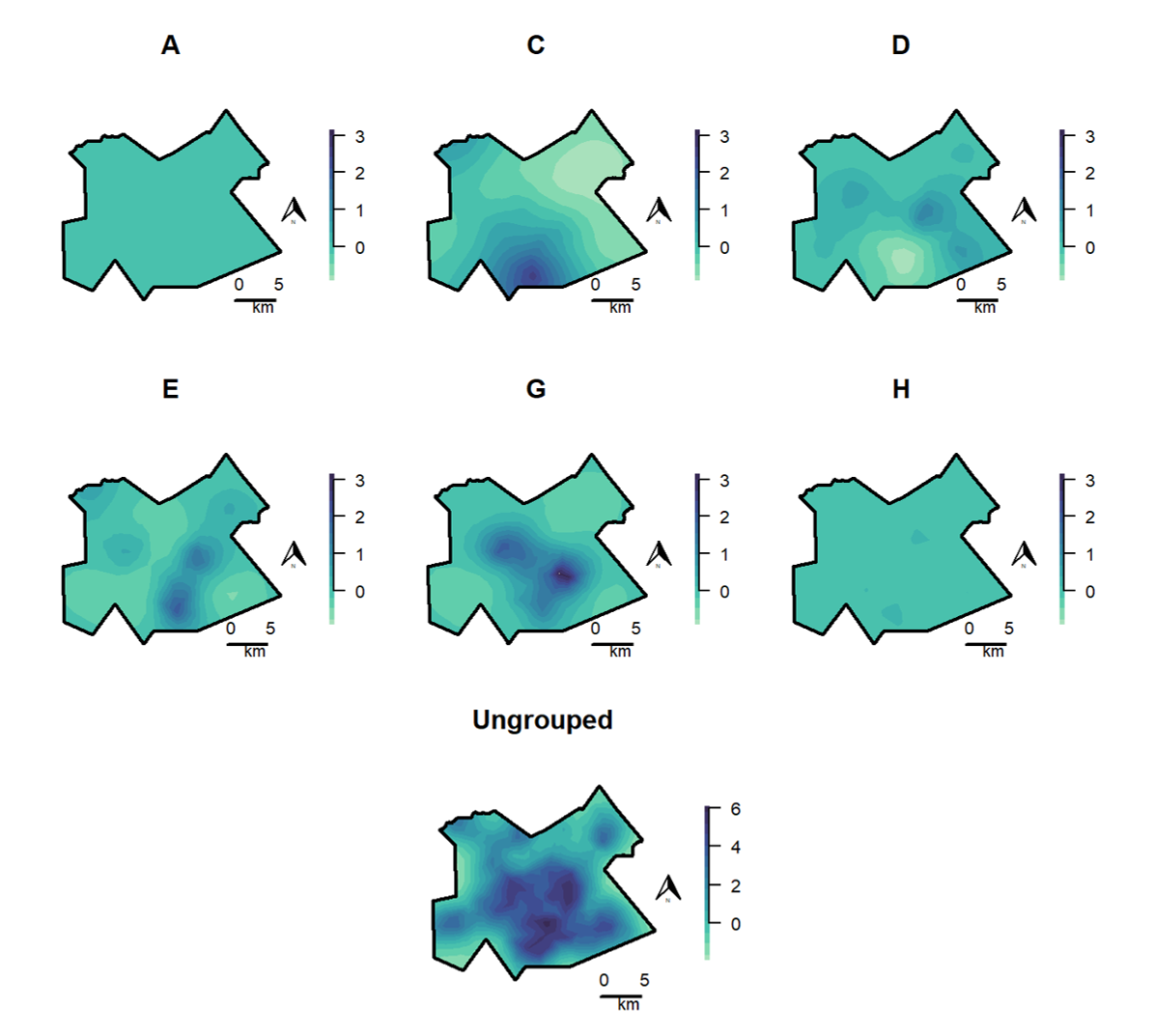
**

**Supplementary Figure 9. Predicted mean spatial intensity of activity spaces for participants in each outbreak group and ungrouped participants using an alternate spatial jitter, Gaborone, Botswana, 2012-2016.** Values are displayed on the same color scale for all outbreak groups, though on a separate color scale for ungrouped controls due to difference in sample size.

**
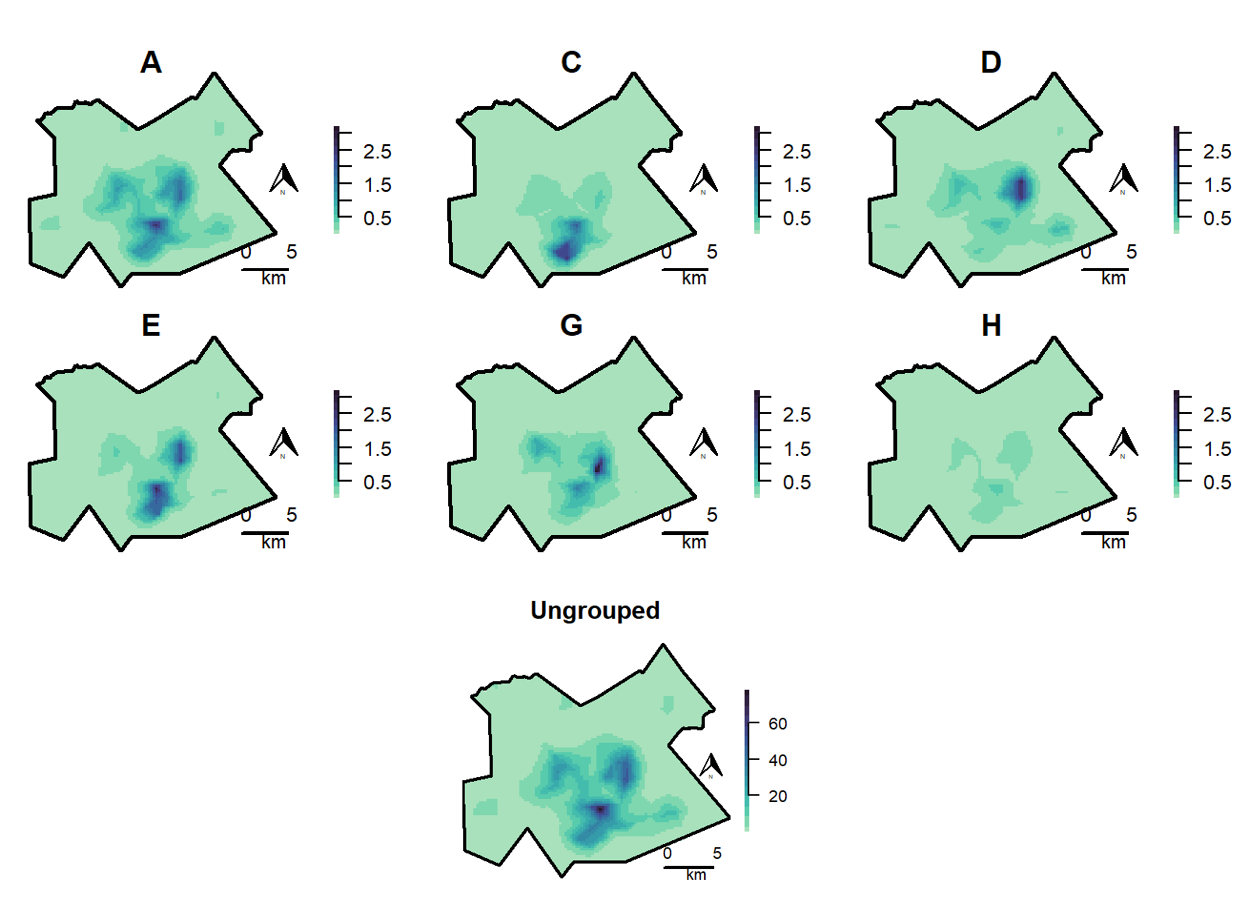
**

**Supplementary Figure 10. Posterior mean estimates of spatial random effect for each outbreak group and controls (ungrouped participants) using 12 SNP threshold, Gaborone, Botswana, 2012-2016.** Darker colored areas indicate places where the estimated spatial effect for each group was increased after accounting for controls. Values are displayed on the same color scale for all outbreak groups, though on a separate color scale for ungrouped controls due to difference in sample size. There are potential areas of overlap between alternate 12-SNP groups and original groups including alternate group B12 and original group C in the central south; alternate group E12 and original group D in a northwest-southeast band; alternate group H12 and original group G in the central west; and alternate group I12 and original group E in the central south and central east. Alternate groups A12 and D12 had relatively broad areas of increased spatial effect suggesting that compared to controls, group A12 was generally more concentrated toward the center of the study area and group D12 was more concentrated toward the east. Groups F12, G12, and J12 had very minimal areas of increased spatial effect, suggesting these groups mostly followed the spatial distribution of controls.

**
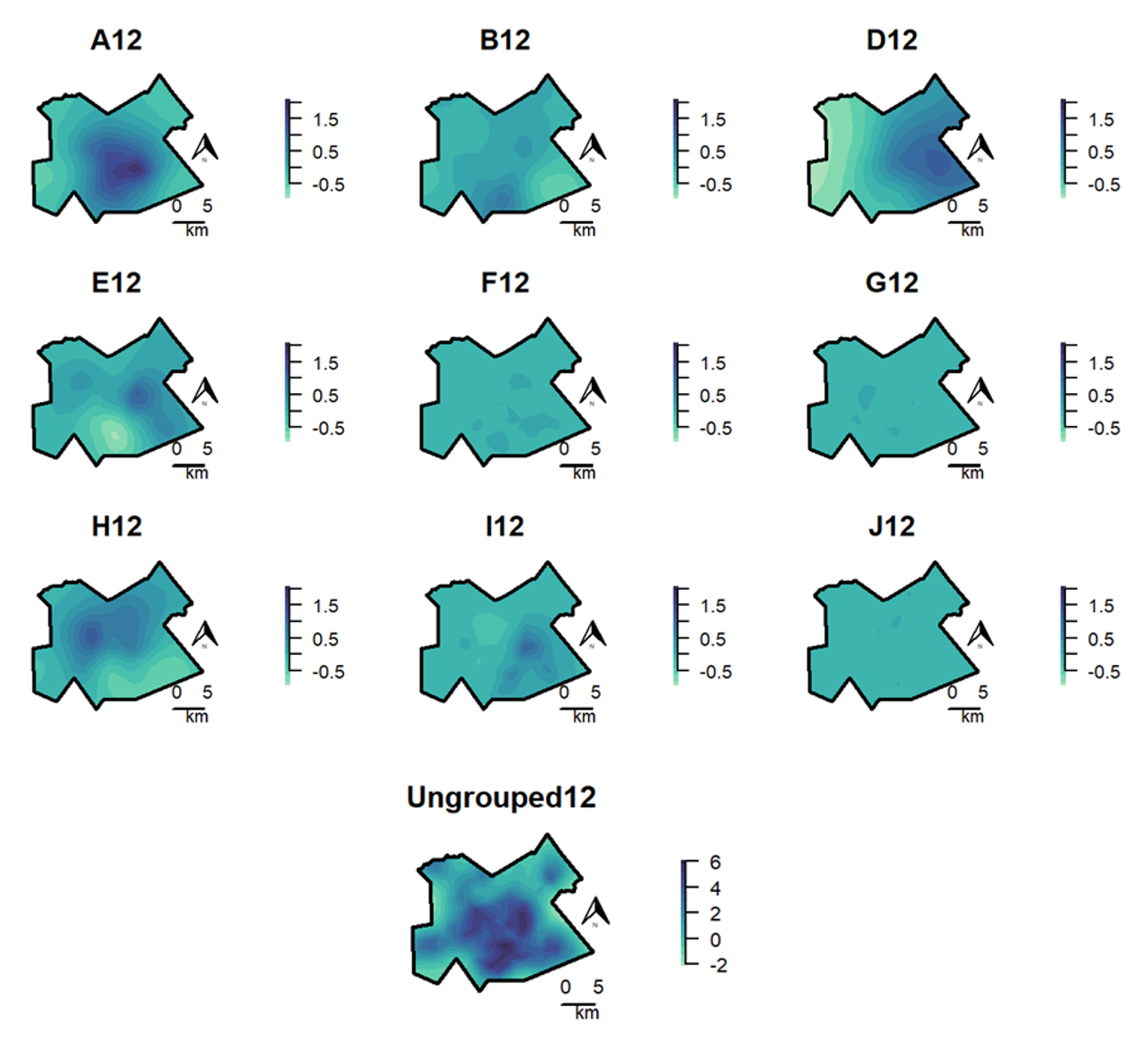
**

**Supplementary Figure 11. Predicted mean spatial intensity of activity spaces for participants in each outbreak group and ungrouped participants using 12 SNP threshold, Gaborone, Botswana, 2012-2016.** Values are displayed on the same color scale for all outbreak groups, though on a separate color scale for ungrouped controls due to difference in sample size. Alternate group B12 was similar to controls but had a relatively pronounced area in the central south, potentially overlapping with highest intensity area for original group C. Alternate group E12 and original group D both had their highest intensity areas in the central east; alternate group H12 and original group G had some similar areas of highest intensity in the central east and central west; alternate I12 and original group E had some similar areas of highest intensity central east and south center.


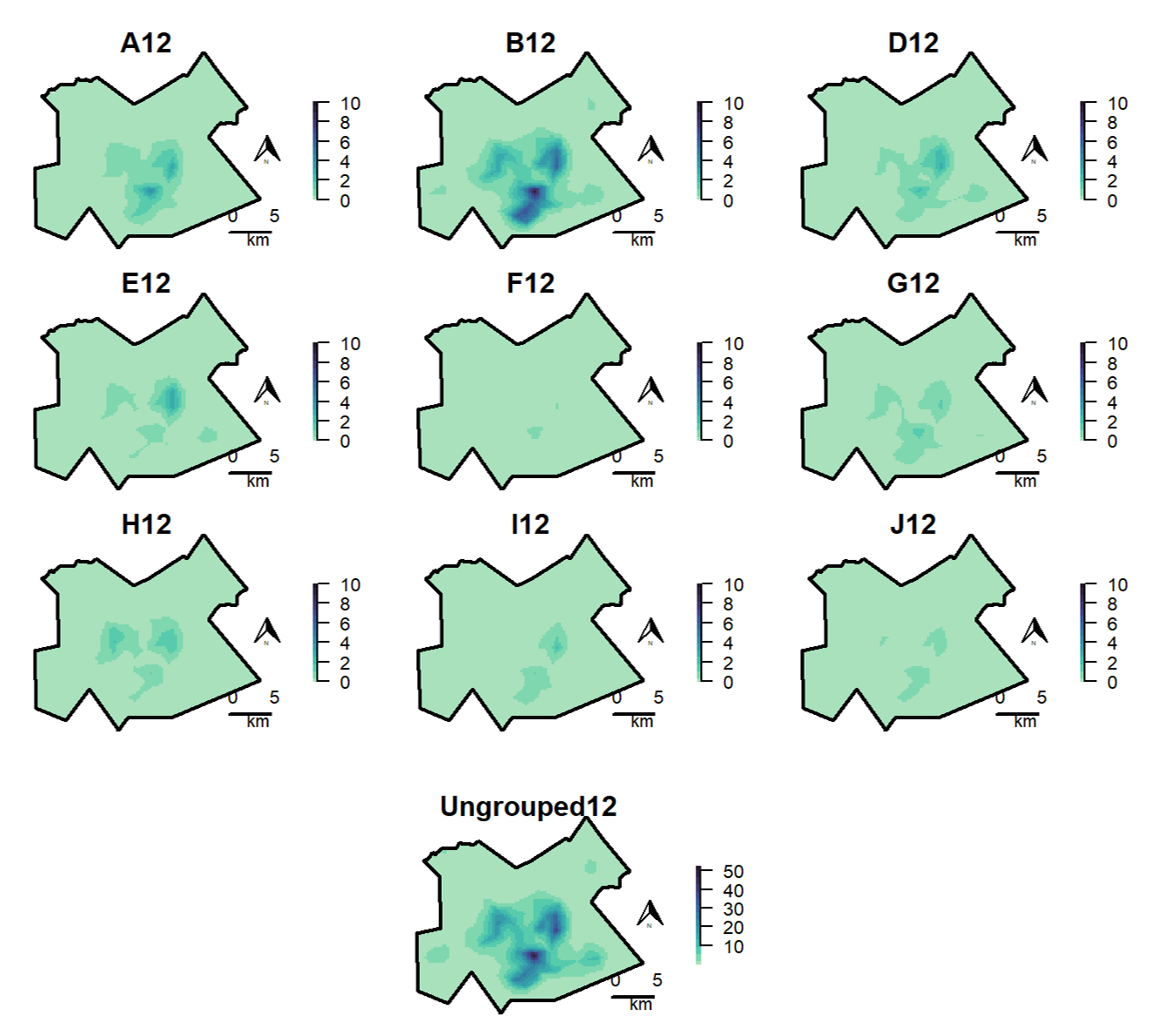

Supplement: Supplementary file 1 — Supplementary Material 1 [file 12942_2026_467_MOESM1_ESM.docx]
